# Stepped vitrification technique for human ovarian tissue cryopreservation

**DOI:** 10.1038/s41598-019-56585-7

**Published:** 2019-12-27

**Authors:** Ellen Cristina Rivas Leonel, Ariadna Corral, Ramon Risco, Alessandra Camboni, Sebastião Roberto Taboga, Peter Kilbride, Marina Vazquez, John Morris, Marie-Madeleine Dolmans, Christiani A. Amorim

**Affiliations:** 1 Pôle de Recherche en Gynécologie, Institut de Recherche Expérimentale et Clinique, Université Catholique de Louvain, Avenue Mounier 52, bte B1.52.02, 1200 Brussels, Belgium; 2 Departament of Biology, Institute of Biosciences, Humanities and Exact Sciences, São Paulo State University (UNESP), Rua Cristóvão Colombo, 2265, Jardim Nazareth, 15054-000 São José do Rio Preto, Brazil; 3 Centro Nacional de Aceleradores (CNA), University of Seville, Calle Thomas Alva Edison 7, 41092 Seville, Spain; 4 Engineering School of Sevilla, University of Seville, Camino Descubrimientos S/N, Isla Cartuja, 41092 Seville, Spain; 5 Service d’Anatomie Pathologique, Cliniques Universitaires Saint-Luc, Avenue Hippocrate 10, 1200 Brussels, Belgium; 6 General Electric Healthcare, Sovereign House, Vision Park, Cambridge CB24 9BY United Kingdom; 7 Gynecology and Andrology Department, Cliniques Universitaires Saint-Luc, Avenue Hippocrate 10, 1200 Brussels, Belgium

**Keywords:** Infertility, Translational research

## Abstract

The advantage of stepped vitrification (SV) is avoiding ice crystal nucleation, while decreasing the toxic effects of high cryoprotectant concentrations. We aimed to test this method for human ovarian tissue cryopreservation. Ovarian cortex was taken from 7 fertile adult women. Samples were subjected to an SV protocol performed in an automatic freezer, which allowed sample transfer to ever higher concentrations of dimethyl sulfoxide (DMSO) as the temperature was reduced. Histological evaluation of the vitrified-warmed tissue showed large numbers of degenerated follicles after 24 hours of *in vitro* culture. We therefore evaluated DMSO perfusion rates by X-ray computed tomography, ice crystal formation by freeze-substitution, and cell toxicity by transmission electron microscopy, seeking possible reasons why follicles degenerated. Although cryoprotectant perfusion was considered normal and no ice crystals were formed in the tissue, ultrastructural analysis detected typical signs of DMSO toxicity, such as mitochondria degeneration, alterations in chromatin condensation, cell vacuolization and extracellular matrix swelling in both stromal and follicular cells. The findings indicated that the method failed to preserve follicles due to the high concentrations of DMSO used. However, adaptations can be made to avoid toxicity to follicles caused by elevated levels of cryoprotectants.

## Introduction

These days, human fertility preservation should be considered a complement of cancer treatments^1^. Indeed, thanks to substantial improvements in treatment and prognosis, ovarian tissue cryopreservation is a technique that has been gaining ground^2^, not only to safeguard fertility, but also to restore hormone levels in women subjected to gonadotoxic treatments or in the menopause^3^. Results obtained so far are promising, with more than 130 babies born to date, and the technique is now on the verge of becoming a possible routine approach and no longer experimental^4^.

Ovarian tissue obtained from these patients is a very precious source of germ cells for fertility preservation, so assays to improve its manipulation have been constantly under development. Slow-freezing (SF) is the most common method used for human ovarian tissue cryopreservation today^5^, derived from a protocol described several decades ago for sheep ovarian tissue^6^.

The main challenge faced during freezing is formation of ice within the tissue, as reducing the temperature leads to ice crystal formation due to ice nucleation. This phenomenon is followed by crystal growth when the molecular arrangement of water is altered to a solid pattern^7^. When the temperature falls very fast, there is no time or energy for molecular rearrangement, so there is no ice crystal formation; the natural disorder of liquid molecules in tissue is maintained, mitigating any disturbance to the system. This forms the basis of the vitrification concept, as the absence of crystals prevents cell membrane damage normally caused by their mechanical activity. While vitrification protocols are already well established for oocytes^8^ and embryos^9^, tissue vitrification remains a challenge because of its variety in composition and size.

Vitrification procedures have been optimized by increasing the viscosity of the solution, which lowers the melting temperature (MT) of the sample and results in its dehydration. In normal conditions, if temperatures drop before the MT is reached, ice formation occurs. However, if the liquid viscosity is increased, crystal formation is avoided^7,10^. On the other hand, an increase in viscosity requires high concentrations of cryoprotective agents (CPAs) in order to extract water from the tissue, which may create other problems, namely toxic effects suffered by cells^11^.

To avoid this issue, samples can be subjected to a low temperature environment, with low rates of potential energy and cell metabolism. Consequently, molecules become less mobile and more stagnant, but remain disordered. Based on work first described by Farrant in 1965^12^, a procedure known as liquidus tracking was developed by Pegg *et al*. to cryopreserve cartilage^13^, involving enhancement of CPA concentrations in samples while decreasing their temperature^14^. This equilibrium procedure prevents ice crystal formation (thanks to high concentrations of CPAs) and decreases cell susceptibility to toxic effects (thanks to low temperatures). The same technique has been tested in bovine ovarian tissue^15^, yielding good rates of CPA perfusion and morphology maintenance. Nevertheless, species-specific responses may vary, especially in human tissue. The aim of this work was therefore to evaluate the effectiveness of a stepped vitrification (SV) protocol based on the liquidus tracking technique in terms of maintenance of the structural characteristics and morphology of human ovarian follicles.

## Results

### Histological analysis

**Figure 1:**
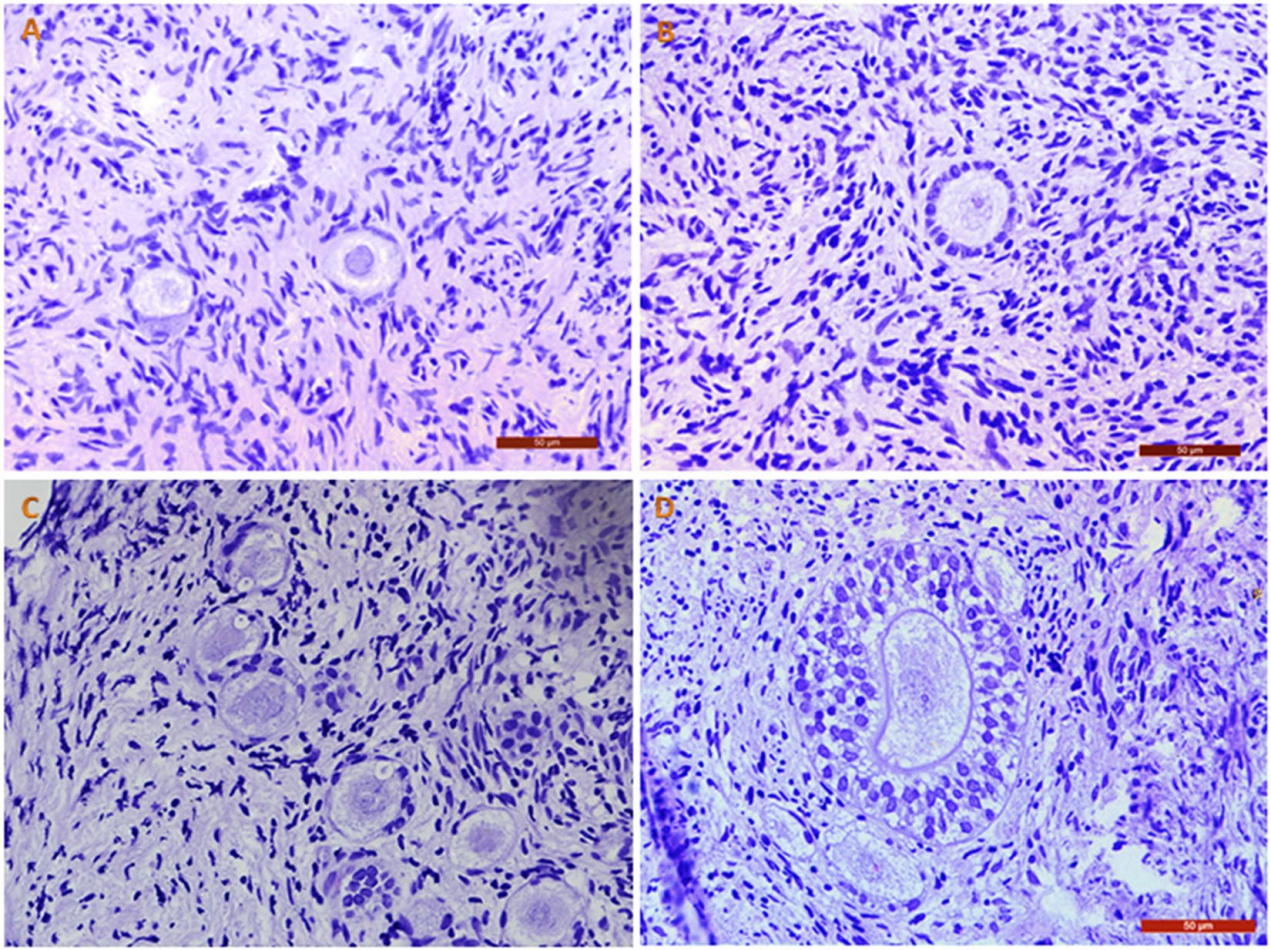
Fresh follicles. Morphologically normal human ovarian follicles observed in fresh ovarian tissue before (**A**,**B**) and after (**C**,**D**) 24 h of IVC. Bars: 50 µm.

**Figure 2:**
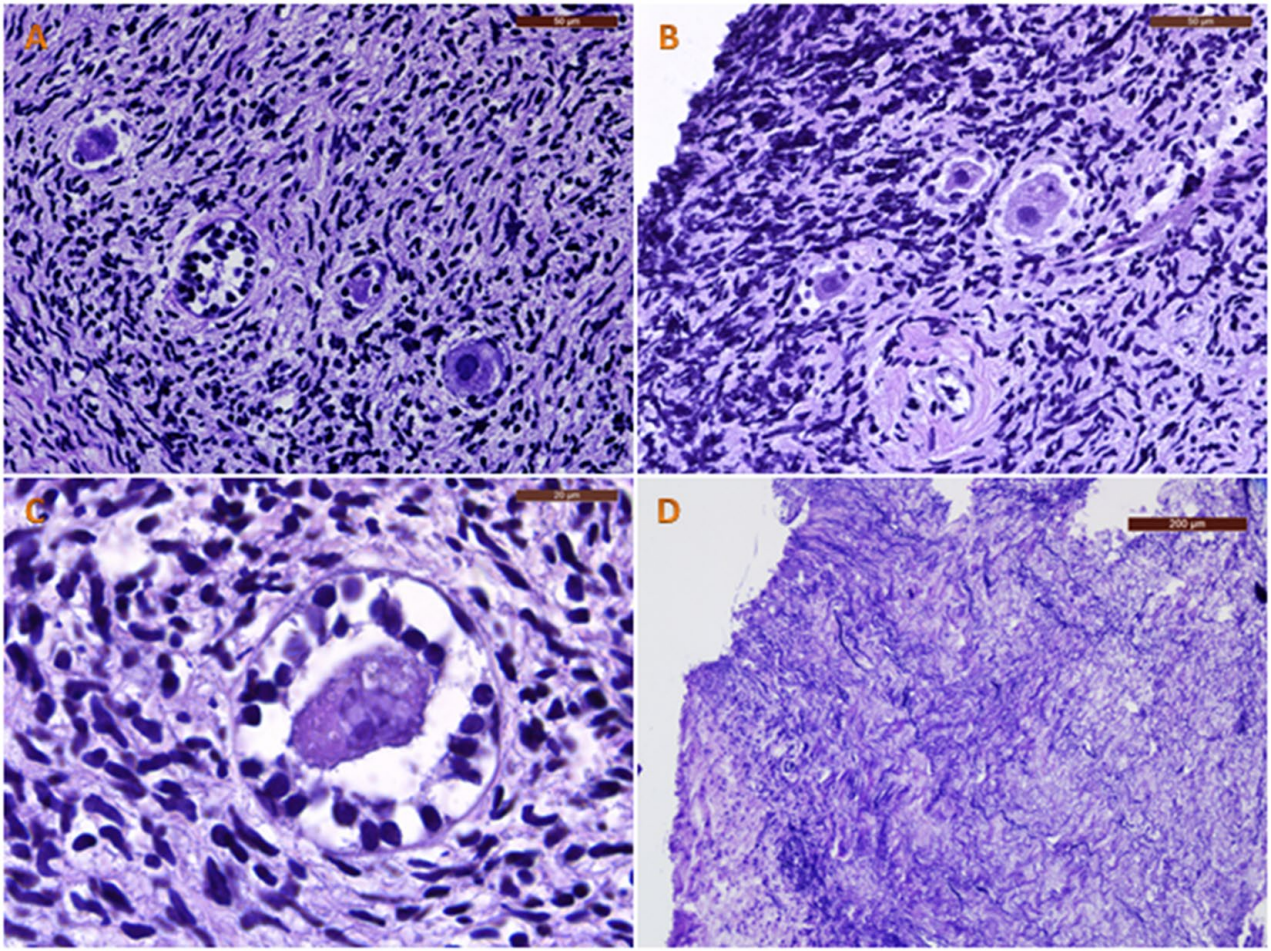
Vitrified-warmed follicles. Human ovarian follicles observed after vitrification-warming and 24 h of IVC. We can identify degenerated follicles with a shrunken oocyte cytoplasm and pyknotic and disorganized granulosa cells (**A**–**C**), as well as fibrotic tissue among stromal cells. (**D**) Bars: 50 µm in A and B; 20 µm in C; 200 µm in D.

Analysis of fresh controls before and after *in vitro* culture (IVC) yielded high percentages of morphologically normal follicles: 98.9 ± 17.7% and 94.5 ± 5.4% respectively. Some of these normal follicles found in our samples are shown in Fig. 1. On the other hand, vitrified-warmed ovarian cortex exhibited signs of degeneration in both follicles and stroma after IVC, as shown in Fig. 2. Unfortunately, 100% of follicles were degenerated. They contained oocytes with strong eosinophilic cytoplasmic staining and shrinkage, and either a pyknotic or absent nucleus, confirming their degenerated status. Granulosa cells also showed signs of pyknosis and were arranged in a disorganized way. In tissue, a large proportion of stromal cells showed pyknotic nuclei and fibrotic deposits.

### X-ray computed tomography

**Figure 3:**
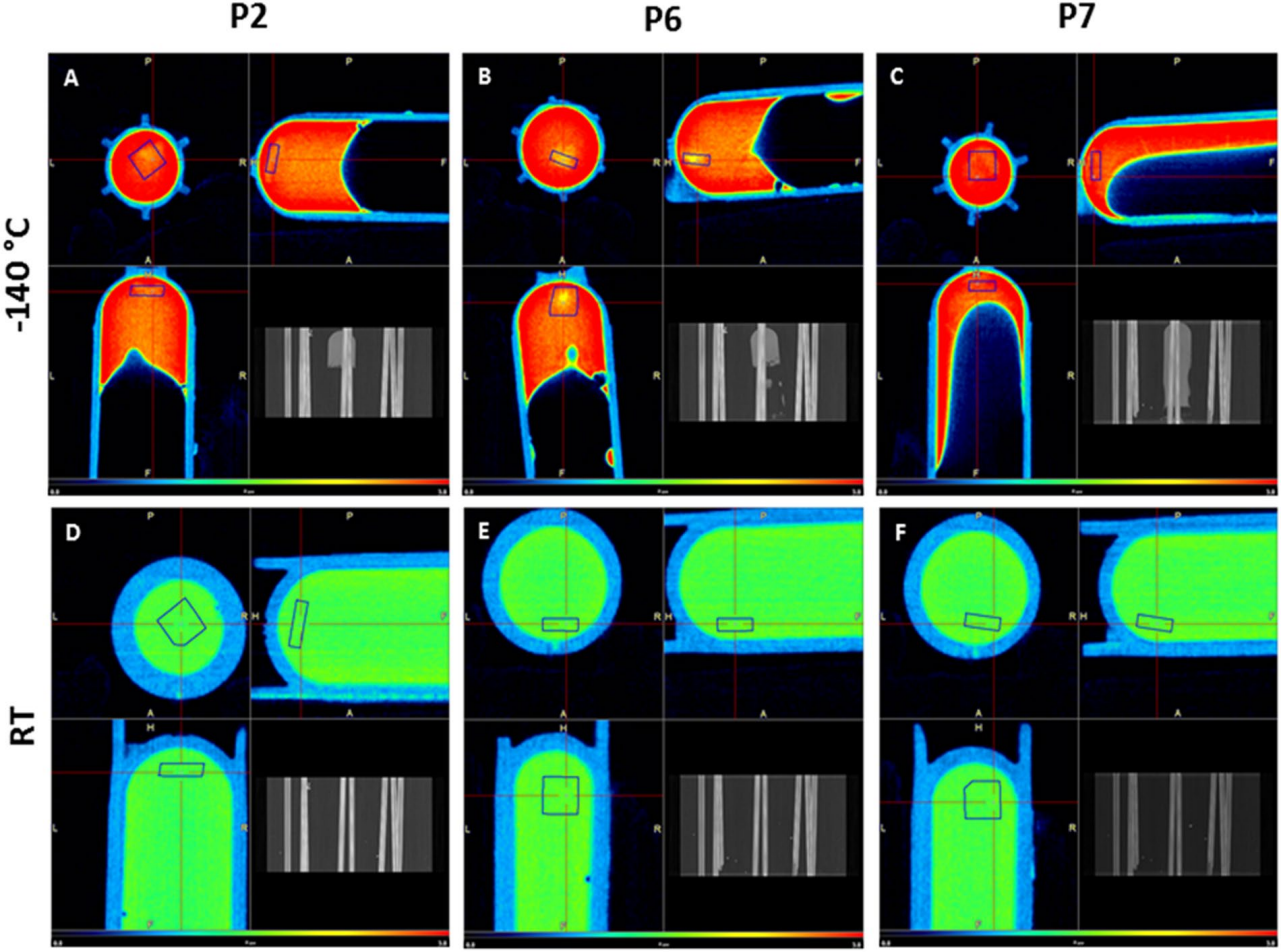
DMSO perfusion assessed by computed tomography. CT images obtained at −140 °C and at RT from three different patients (P2, P6 and P7) showing vitrified and warmed ovarian cortex. Spatial resolution is 0.2 mm and the color scale runs from dark blue for low attenuation (1.2 CT, lower concentrations of DMSO) to intense red for high attenuation (3.0 CT, higher concentrations of DMSO). Blue-colored squares shown in all images are volumes of interest (VOIs) located within the tissue to obtain statistical data on attenuation and DMSO concentrations. Tissue can barely be distinguished from surrounding medium in A and C, while in B it can be partly differentiated, showing less attenuated coloration (yellow) than medium containing 50% DMSO (red). After cryoprotectant removal, there is no difference in attenuation levels in the vials at RT. RT: room temperature.

**Figure 4:**
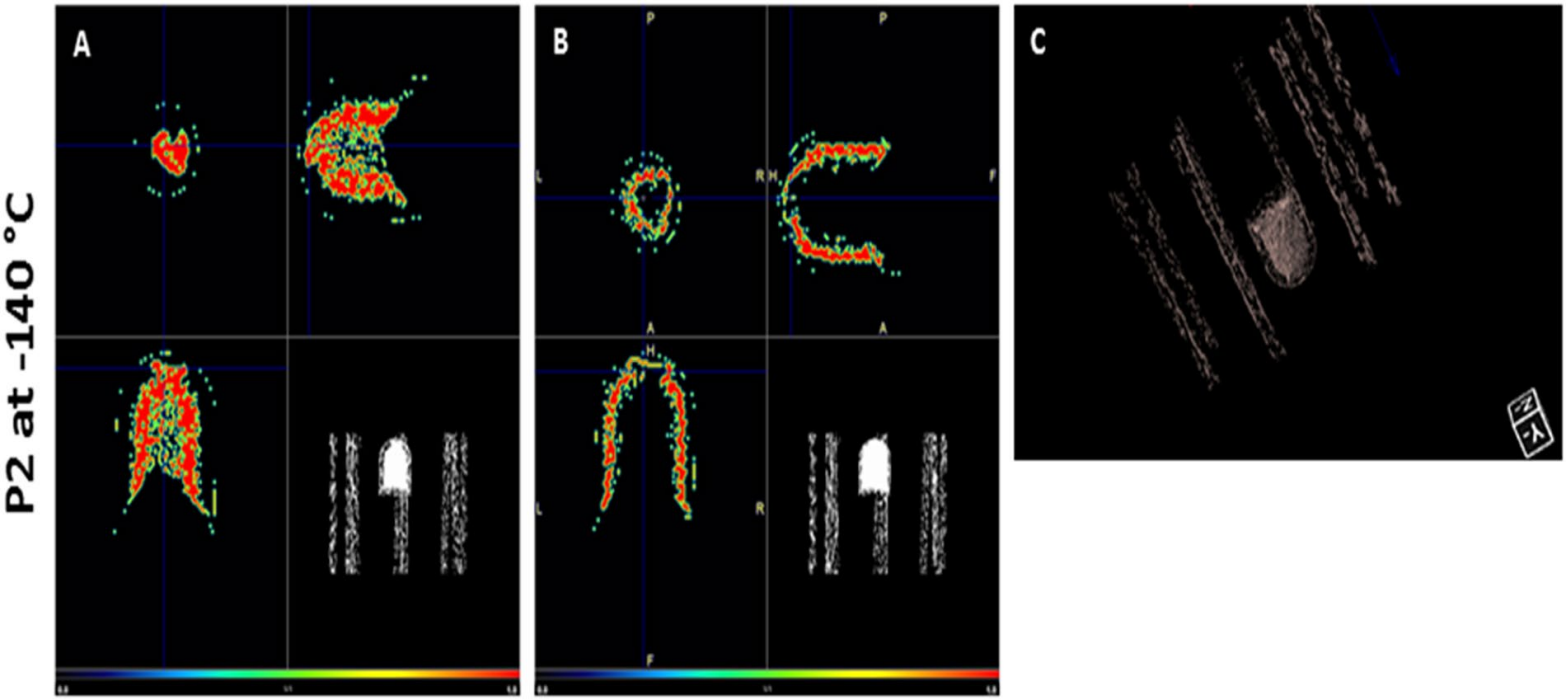
Segmented computed tomography image. Computed tomography image obtained from one sample at −140 °C. (**A**) Segmentation of the CT image showing only part of the picture with a threshold value from 2.6 to 2.8 CT (on a scale of 0.0 to 1.0), which corresponds to a concentration of 38.2% to 44.4% v/v DMSO. The tissue can be distinguished at the bottom of the cryovial with a similar concentration to the medium. (**B**) Image obtained under the same conditions as in A, but with a threshold value from 2.8 to 3.0 CT, which corresponds to a concentration of 44.4% to 50.6% v/v DMSO. Note the effect at the borders, suggesting higher concentrations than the solution in the middle. (**C**) 3D images obtained with the same threshold value as in A. CT: cycle threshold.

Analysis at −140 °C revealed no difference in attenuation between tissue and surrounding medium in two out of three samples evaluated by Nano computed tomography (CT), indicating that the system was well equilibrated (Fig. 3A–C). In one of the samples, although tissue could barely be distinguished from the solution in the inner part of the vial, the outer region showed higher attenuation close to its border (Fig. 4). This constitutes a normal beam hardening effect, which was corrected with CT calibration. Estimated DMSO concentrations in tissues are shown in Table 1.

**Table 1:** Average, minimum and maximum DMSO concentrations (% v/v) in tissues analyzed at −140 °C and RT, calculated from a VOI of 3 × 3 × 1 mm in the tissue, with calibration curves established for each temperature.

Temperature – Patient	Average CT values	Average % v/v DMSO	Min-Max % v/v DMSO	
−140 °C	P7	2.82 (0.10)	45.2 (+0.9)	38.0–55.0
	P2	2.72 (0.14)	42.0 (+0.8)	33.2–53.2
	P6	2.59 (0.18)	38.1 (+0.7)	21.4–53.1
RT	P7	1.49 (0.05)	0.5 (+1.0)	−4.8–7.3
	P2	1.46 (0.05)	−0.8 (+1.0)	−6.2–4.7
	P6	1.49 (0.07)	0.5 (+1.0)	−6.4–8.4

The average CT error is the standard deviation of the 3 × 3 × 1 mm^3^ VOI, while average DMSO concentration errors correspond to the standard error of the mean. RT: room temperature; CT: computed tomography; DMSO: dimethyl sulfoxide.

After the warming curve was achieved and CPA removal concluded, there was no visible difference between X-ray attenuations of tissue samples and surrounding medium containing 0% DMSO at RT (Fig. 3D–F). This attenuation similarity led us to conclude that CPA removal was adequate after warming.

### Freeze-substitution

**Figure 5:**
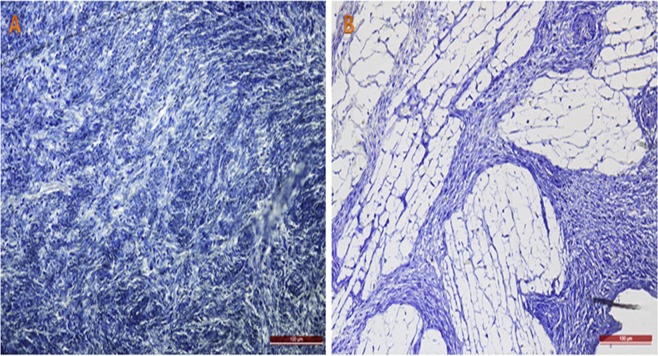
Ice crystal formation assessment. Ovarian tissue fixed by freeze-substitution. Note the absence of spaces between cells and fibers in human ovarian cortex subjected to the stepped vitrification procedure (**A**), compared to control ovarian tissue subjected to an inefficient cryopreservation procedure. (**B**) Toluidine blue staining; 200x magnification; bars: 100 µm.

Human ovarian tissue fixed at low temperatures after equilibrium vitrification showed no signs of ice crystal formation, confirmed by normal attachment between cells and no empty spaces in the tissue (Fig. 5A). In sheep ovarian tissue cryopreserved using an inefficient technique (Fig. 5B) and serving as a control for comparative purposes, we detected large empty spaces corresponding to ice crystals formed during the freezing procedure.

### Transmission electron microscopy

Follicles (n = 3) from fresh and vitrified-warmed tissue from three patients were localized and analyzed by TEM. They were all at primordial or primary stages of development.

**Figure 6:**
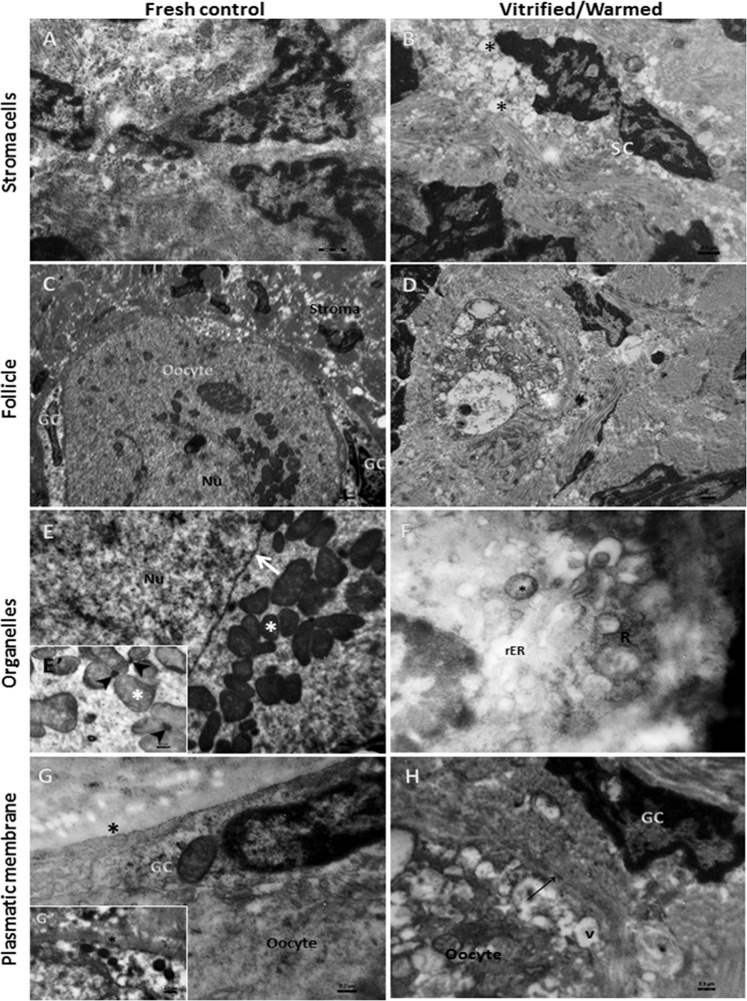
Transmission electron microscopy. TEM pictures from fresh and vitrified-warmed ovarian tissue. (**A**) Good preservation of stromal cells (3000x); (**C**) Good morphological preservation of cells and attachment between the oocyte and granulosa cells, and the follicle and stroma, in a primordial follicle (700x). (**E**) Well preserved nuclear envelope (arrow) and accumulation of normal mitochondria (white asterisks) around it (7000x). (**E’**) Group of mitochondria attached to a dense amorphous granule (arrowheads). G, (**G’**) Preservation of oocyte and granulosa cell membranes, with the presence of microvilli and the basal membrane (black asterisks) (4000x). (**B**) Stromal cells showing the presence of several vacuoles in their cytoplasm (asterisks) and a nucleus with highly condensed chromatin (3000x). (**D**) Poorly preserved follicle and an oocyte nucleus with a pyknotic aspect and highly condensed chromatin (400x). (**F**) Presence of degenerated mitochondria (asterisk) and rough endoplasmic reticulum (rER) close to an apparent group of ribosomes (R) (4000x). (**H**) Presence of vacuoles (v) and signs of cell membrane degeneration in the oocyte (arrow) (4000x). SC: stromal cells; GC: granulosa cells; Nu: nucleus.

Fresh tissue contained stromal cells of varying shape, usually with moderately dispersed chromatin. Among these stromal cells, deposits of collagen fiber beams differed from patient to patient, being thicker and more scattered in one subject. Nevertheless, stromal cells from all patients showed signs of morphology maintenance (Fig. 6A). Follicles had a well preserved oocyte (Fig. 6C) with numerous mitochondria of normal shape (round or cylindrical) and electron density, usually located close to the nuclear envelope (Fig. 6C,E) and frequently surrounding a small, dense, amorphous granule (Fig. 6E’). Some lysosomes and small lipid droplets were also present. The cytoplasm exhibited a uniform granular background (Fig. 6E,G), while nuclei showed dispersed heterochromatin and a well preserved nuclear envelope (Fig. 6E). Adequate attachment was maintained between granulosa cells and oocytes, as well as microvilli (Fig. 6G). A continuous and intact basal membrane layer between the granulosa and stromal cells evidenced the expected appropriate follicle morphology (Fig. 6G,H).

Since no preserved follicles were found by light microscopy after IVC, we decided to analyze ovarian tissue ultrastructure immediately after warming in order to identify any alterations typically caused by cryopreservation. Although cell metabolism and activity were not entirely reestablished, clear signs of degeneration were already visible in cell ultrastructure. It is important to state that during analysis of semi-thin sections by toluidine blue staining under light microscopy, it was difficult to detect signs of degeneration (200x magnification).

Stromal cells showed condensed chromatin, poorly preserved organelles and numerous vacuoles in their cytoplasm (Fig. 6B). Oocyte organelles were not preserved (Fig. 6D) and displayed enlarged rough endoplasmic reticulum cisternae, either with flocculent content or in the form of a clear vacuolar structure, sometimes surrounded by detached clustered ribosomes (Fig. 6F). Mitochondria exhibited clear signs of degeneration, such as disrupted shape, slightly clarified matrices and the remains of cristae (Fig. 6F). Large vacuoles were also observed throughout the cytoplasm, as was degeneration of cell membranes in terms of detachment and disintegration of the lipid bilayer (Fig. 6H). The nucleus consistently showed a dense and disorganized chromatin structure, with degeneration of the nuclear envelope and clear signs of pyknosis (Fig. 6D).

## Discussion

In this study we evaluated a new vitrification procedure for cryopreservation of human ovarian tissue. However, results from histological analysis were initially discouraging, since no normal follicles were found. We therefore investigated, examining possible reasons why follicle structure was not maintained. Since the main obstacles encountered during cell cryopreservation are ice crystal formation and CPA toxicity, subsequent evaluations were directed at these two possibilities.

CT analysis found DMSO concentrations to be adequate, and the morphological appearance of ovarian tissue after freeze-substitution confirmed no ice crystal formation. A number of alterations to the ultrastructure of follicles were identified by TEM, however revealing that CPA toxicity was responsible for the lack of normal follicles.

Although SF is the most commonly adopted protocol for human ovarian cryostorage today, there is great interest in vitrification systems. Superior results have indeed been obtained with vitrification compared to SF in terms of follicle viability^16^, DNA fragmentation, proportion of intact preantral follicles^17^, preservation of stromal cells^18^, and follicle density and activation rates^19^. A recent review and meta-analysis^20^ concluded that vitrification may well be more effective than SF, but the authors stressed that variations in protocols could negatively impact the dissemination of the technique and its application in routine clinical practice. It is, therefore reasonable to wonder why a preference to SF still persists. One possible answer is that in addition to the threat of CPA toxicity, direct tissue contact with non-sterile liquid nitrogen could deter practitioners from attempting vitrification^21^. However, this second issue may be avoided by using the Cryotop method^22^, pooled straws^23^ or even cryotubes, as described in the present work, so CPA toxicity remains the main concern.

The effects of this toxicity led us to consider the alternative of SV in humans, which was recently successfully tested for the first time in ovarian tissue^15^. While results obtained in bovine tissue were promising in terms of follicle morphology preservation, this was not the case in the present study. One of the reasons for the discrepancy may have been that Corral *et al*. did not proceed to IVC after warming, aware that omission of this step could impair recognition of organelle changes due to toxicity. It is noteworthy that tissue fixed right after warming in the present study did not show obvious signs of degeneration either when analyzed by light microscopy (200x magnification with toluidine blue staining), but it was detected upon ultrastructural analysis. This confirms the need for an incubation step of at least 24 hours of IVC to evidence the impact of the SV method on follicle viability by histology, as previously reported^24^. The opposite findings could also be explained by the heterogeneity in plasmatic membrane composition between different species and types of cells^25^, since in articular cartilage^13^ and encapsulated liver cells^26^, for example, the SV protocol proved extremely efficient at maintaining cell viability and functionality.

During cryopreservation, ice nucleation occurs when the MT is reached, followed by its growth in water molecules, which rearrange themselves in an orderly manner aiming to form a solid crystal^7^. This rearrangement happens wherever water molecules are located, easily destroying tissue components. However, for vitrification to proceed properly in a stepped way, solutes in the systems need to be concentrated/viscous enough to inhibit nucleation^27^. It is known that at −40 °C, the viscosity of highly concentrated DMSO solution is increased, while the permeability and fluidity of membranes are decreased^28^, which may impair CPA perfusion. Nevertheless, our CT results showed average equilibrium percentages of DMSO to be between 38% and 45%. According to Wowk (2010), such DMSO concentrations in tissue ensure an absence of ice, preventing crystal nucleation at −40 °C and promoting formation of a vitreous state at −150 °C, the temperature of liquid nitrogen vapors^7^. This assumption was corroborated by the freeze-substitution method; since no empty spaces were detected in the ovarian tissue, we had confirmation of adequate CPA equilibrium that promoted appropriate externalization of water and viscosity of the system.

Another important finding was the absence of DMSO in samples after warming. Remains of CPA in tissue have raised concerns because of their potential for damage, such as impacts on epigenetic profiles, as observed in embryoid bodies^29^. After evaluation of residual DMSO in warmed and thawed ovarian tissue, DMSO remnants were only found in vitrified samples^30^, which authors attribute to the fast warming protocol. Since warming was performed slowly in the present study, we achieved total of CPA removal, anticipating no long-term toxic effects on either the potential host or potential embryo.

Once high concentrations of DMSO had been reached and both dehydration and viscosity of compounds deemed adequate, and despite the temperature reduction scheme in the SV method being intended to prevent CPA toxicity, follicle death still ensued. This led us to attribute cell death to chilling injury or high DMSO levels. According to Mazur *et al*. (1992), chilling injury is thought to increase with exposure time to very low temperatures^31^ and its effects are based on membrane phase transition modifications causing membrane disintegration. Reduced CPA toxicity under low temperature condition is related to enzyme activity decreases and lower reaction kinetics in general, be they chemical or osmotic stresses^26^. Since the toxic effects seen in the present study were related to membrane degeneration, they were probably caused by chemical changes in the phospholipid chain conformation that is not attenuated by low temperatures.

Thanks to its capacity to easily cross cell membranes, DMSO is often used in living systems for enhanced penetration of drugs and cryoprotection. It acts by means of three different mechanisms depending on its concentration: (i) by decreasing membrane thickness when used in the range of 2.5–7.5 mol%; (ii) by inducing formation of transient water pores in the range of 10–20 mol%; or (iii) by permanently disorganizing the bilayer structure at concentrations above 25 mol%^32^. The cryoprotective properties of DMSO rely on membrane pore formation for externalization of water, but the membrane is unable to recover in concentrations over 25%, even at low temperatures^33^, as seen in the present study.

Because the toxic effects of DMSO are mostly associated with cell membrane damage^34^, we assessed toxicity by ultrastructural analysis, which is a widely applied technique^35–37^ that allows cell membrane evaluation. It revealed extensive damage to organelles and plasmatic membranes. After DMSO penetrates cells, it is distributed throughout the cytoplasm via a microcirculatory tubule system in a directed manner^38^, enabling its spread through cell contents and dissemination to organelles. Mitochondrial alterations were indeed evident. According to Eyden *et al*. (2004), mitochondria and rough endoplasmic reticulum are the most sensitive organelles in oocytes subjected to cryopreservation^39^. Cell vacuolization was consistently observed during our analysis and is widely reported in the literature; it may be related to swelling and coalescence of smooth endoplasmic reticulum and the Golgi apparatus or plasmatic membrane invaginations due to endocytic vesicles^40–42^. Irregularly shaped oocyte nuclei and chromatin thickening were also encountered after ovarian tissue cryopreservation^36^. These TEM findings corroborate observations of DMSO toxicity described in the literature: cell membrane disintegration and apoptosis induction^34^.

In conclusion, our results suggest that rather than preserving human ovarian tissue, 50% DMSO at −40 ^o^C causes degeneration of preantral follicles and stromal cells, the concentration being too high. One way of solving this problem could be lowering DMSO levels in the final vitrification solution or even associating CPAs, all at lower concentrations. Indeed, since our objective is to increase viscosity and promote externalization of water from cells, this approach could mitigate membrane disintegration induced by 50% DMSO^33^.

## Methods

### Ethics and experimental design

Ovarian tissue was taken from 7 women (27–33 years of age) undergoing laparoscopic procedures for benign gynecological disease (six patients with endometriosis and/or endometriomas and one with low-grade endometrial stromal sarcoma) after obtaining informed consent. Use of human ovarian tissue was approved by the Institutional Review Board of the Université Catholique de Louvain on November 28, 2016 (IRB reference 2012/23MAR/125, registration number B403201213872). All methods were performed in accordance with the relevant guidelines and regulations.

After removal, ovarian biopsies were immediately transported to the laboratory in Dulbecco’s Modified Eagle Medium (DMEM/F12; Gibco, USA) in sterile and low temperature conditions (0 °C). Once in the laboratory, the tissue was washed and cleaned of its medullary part, before being cut into 5 × 5 × 1 mm pieces and subjected to the previously described vitrification procedure^15^. Our experiments did not investigate conventional freezing of ovarian tissue, as this has been widely documented in the literature (Table 2).

**Table 2:** Summary of the effect of conventional freezing on ovarian tissue compared to our findings after equilibrium vitrification.

Parameter	Reference	Results	Our findings
Histological analysis	^46^ (human OT)	97% intact follicles	No intact follicles were found.
	^47^ (human OT)	81% intact follicles	
	^48^ (human OT)	68% intact follicles	
	^19^ (human OT)	81% intact follicles	
X-ray computed tomography	^49^ (human OT)	Frozen samples: OT could not be distinguished from the surrounding solution, although the presence of ice was less pronounced in the sample area. Average DMSO concentration: 11% Thawed samples: effective removal of DMSO.	Frozen samples: no difference observed in attenuation between tissue and surrounding medium in two out of three samples, indicating that the system was well equilibrated. Average DMSO concentration: 38.1–45.2%. Thawed samples: average DMSO concentration: −0.8% to 0.5%.
	^50^ (bovine OT)	Frozen samples: average DMSO concentration: 7.5%	
	^51^ (bovine OT)	Very small ice crystals observed at similar proportions throughout the whole vial, including the area where the tissue was located.	
Freeze-substitution	^52^ (rabbit OT)	Frozen samples: densely packed and well delimited cellular material, but faster cooling rates caused more extensive disruption and shrinkage of tissue elements. Vitrified samples: no ice formation.	No sign of ice crystal formation, confirmed by normal attachment between cells and no empty spaces in the tissue.
	^53^ (sheep OT)	Frozen samples: widespread presence of large and small ice crystals throughout the extracellular matrix. Vitrified samples: no sign of ice formation.	
Transmission electron microscopy	^54^ (human OT)	Cytoplasmic vacuolization of GCs, oocyte, and fibroblasts. Microvilli between oocyte and GCs. Well preserved chromatin, basal membranes, gap junctions, and cell membranes. Normally organized collagen bundles.	Poorly preserved organelles: enlarged rough endoplasmic reticulum with detached and clustered ribosomes, mitochondrial degeneration, large vacuoles in the cytoplasm, detachment and disintegration of the lipid bilayer of cell membranes, disorganized chromatin, pyknosis.
	^55^ (human OT)	Intact follicles with no morphological deformation.	
	^56^ (human OT)	Oocytes with regularly shaped nuclei and finely dispersed chromatin. Mitochondria with moderately electron-dense matrices and curved cristae. Well preserved stromal cells.	
	^18^ (human OT)	Oocytes with prominent nucleoli, normally distributed euchromatin, and well preserved nuclear envelopes. Dense and well preserved mitochondrial matrices, well preserved GCs.	
	^57^ (human OT)	Healthy-looking follicles: large vesicular nuclei with dispersed chromatin, mitochondria with low-density matrices and few peripheral cristae, close interdigitations between oocyte and GCs.	

OT: ovarian tissue; GCs: granulosa cells.

Small fragments (1 × 1 × 1 mm) were used as fresh controls and immediately fixed for analysis by light and transmission electron microscopy (TEM). Short-term (24 hours) IVC of fresh ovarian tissue (1 × 1 × 1 mm^3^) was also performed for control purposes to ensure culture protocol effectiveness for cell morphology maintenance and activity. All mentioned protocols are described below.

All chemicals used were purchased from Sigma-Aldrich, unless otherwise stated.

### Vitrification procedure

A programmable controlled-rate freezer was used (Asymptote, General Electric Healthcare, VIA Freeze Research, Sovereign House, Cambridge, UK). It was equipped with an SV device designed to move samples to vials with different concentrations of dimethyl sulfoxide (DMSO).

Four 3 ml vials were filled with minimal essential medium (MEM + GlutaMAX; Gibco, USA) containing increasing concentrations of DMSO (10%, 20%, 40% and 50%), 8% human platelet lysate (Macopharma, France) and 2 IU/ml heparin (LEO Pharma, Denmark). The vials were then placed on the cooling plate of the freezer and remained open throughout the vitrification procedure, so tissue samples could move freely from one vial to another (Fig. 1). The entire procedure was performed inside a laminar flow hood under sterile conditions.

**Figure 7:**
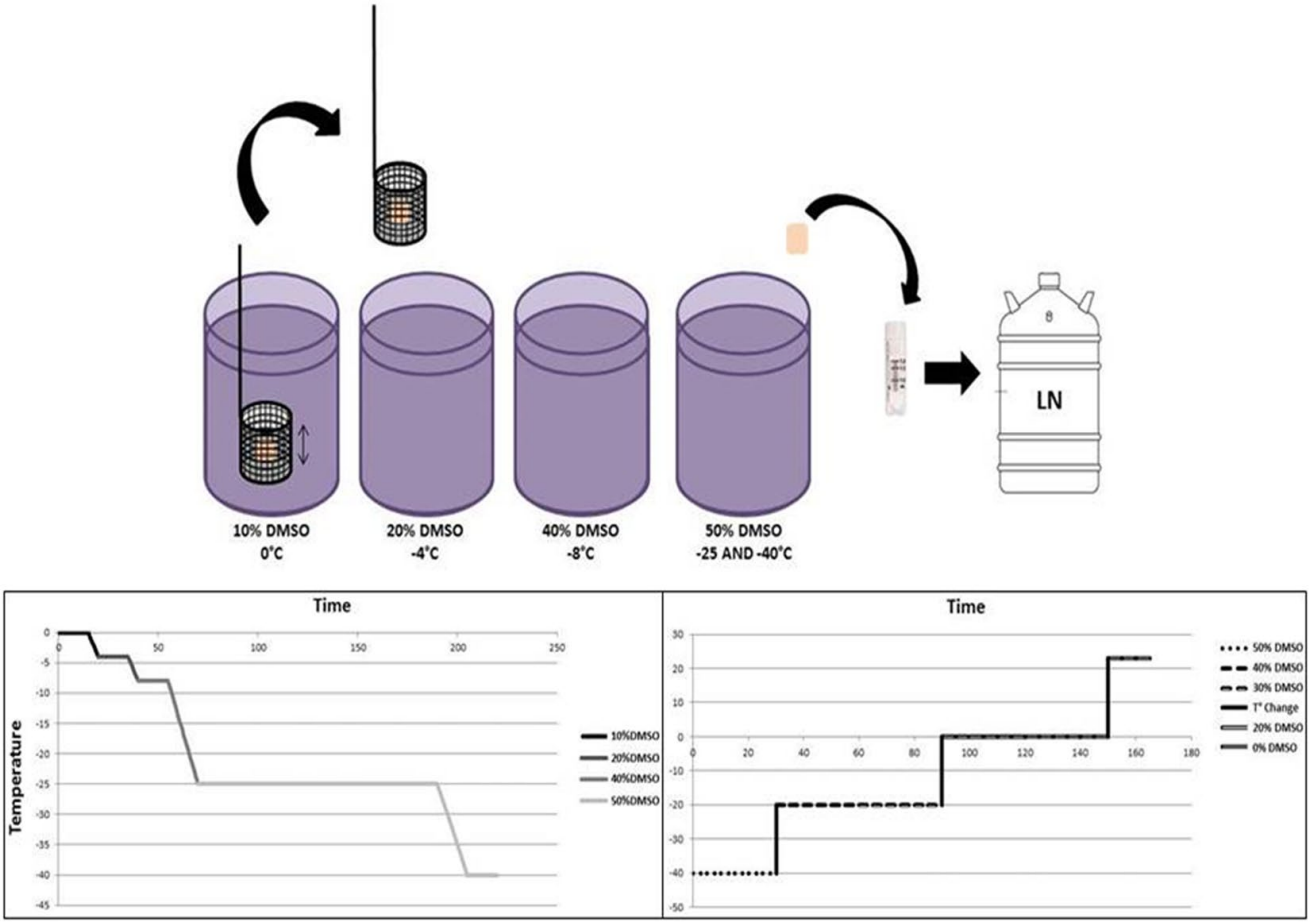
Equilibrium vitrification system. Cooling and warming curves achieved in the programmable freezer with the stepped vitrification device. DMSO: dimethyl sulfoxide; LN: liquid nitrogen.

The samples were subjected to DMSO permeability in low temperatures, as demonstrated by the curve shown in Fig. 7. During each bath, ovarian tissue was kept submerged in medium for different periods of time, and moved in a constant up-down motion inside a small plastic basket in order to improve CPA perfusion, promoting faster equilibration of the tissue. In short, the ovarian cortical tissue was subjected to a 10% v/v DMSO bath at 0 °C. The samples were then transferred to ever higher concentrations of DMSO concurrently with a temperature drop in the machine (20% DMSO at −4 °C, 40% DMSO at −8 °C, 50% DMSO at −25 °C). Finally, the temperature was reduced to −40 °C and the samples were placed in cryovials containing pre-cooled (−40 °C) 50% DMSO solution to proceed with vitrification. Temperature monitoring was carried out using a thermocouple data logger deposited inside a side vial containing 50% DMSO solution. The vials were kept in liquid nitrogen vapors (approximately 2 cm above the liquid nitrogen surface) inside a cryotank for 5 minutes and vitrified to −150 °C. They were then submerged and stored in liquid nitrogen until warming.

### Warming procedure

An adapted previously described slow-warming protocol^13^ was applied. Briefly, the cryovials were removed from the liquid nitrogen and placed in the pre-cooled programmable freezer (−40 °C) for 30 minutes. Thereafter, the solution was replaced in order to remove the CPA from the samples. Lower DMSO concentrations and higher temperatures were used, as shown in Fig. 7. The samples were kept in a 40% DMSO solution at −20 °C for 30 minutes, then in 30% DMSO for another 30 minutes. At 0 °C, 20% and 0% DMSO solutions were utilized for 30 minutes each. Finally, three baths without DMSO lasting 5 minutes each were performed at room temperature (RT) (23 °C). The ovarian tissue fragments were then divided into 1 × 1 × 1 mm pieces that were either subjected to 24 hours of IVC, as described below, or fixed for TEM evaluation.

### 24 h *in vitro* culture

Fragments of fresh and warmed ovarian cortex were divided into 1 × 1 × 1 mm pieces and *in vitro* cultured in an attempt to restore the activity of follicles and other ovarian cells, and determine the impact of the cryopreservation procedure on their viability. The basic medium used was the same as that described for vitrification solution enriched with insulin, transferrin and selenium (ITS, Gibco, USA, 1%), pyruvic acid (2 mM), ascorbic acid (50 µg/ml) and antibiotics, namely amphotericin B, penicillin and streptomycin (Gibco, USA, 1%). The cortical pieces were kept inside a 5% carbon dioxide (CO_2_) incubator (Thermo Fischer Scientific, USA) at 37 °C. After 24 hours of culture, they were fixed in 4% paraformaldehyde and processed for histological analysis.

### Histological analysis

After fixation in 4% paraformaldehyde, fresh and cultured samples underwent histological processing (dehydration and clarification) before being embedded in paraffin. The blocks were then subjected to 5-µm-thick sectioning in a microtome and the slides were stained with hematoxylin and eosin for a general analysis of the tissue morphology and follicle counting.

Follicles were classified as morphologically normal when they had an intact spherical aspect, with uniform distribution of granulosa cells and a spherical oocyte showing no retractions or vacuoles attached to the surrounding stroma. In the absence of these characteristics, follicles were considered to be degenerated. Only those exhibiting a visible oocyte nucleus were counted.

### X-ray computed tomography

To determine CPA perfusion and removal efficiency, samples from three different patients were analyzed by computed tomography (CT) in order to ascertain DMSO concentrations in ovarian tissue after vitrification. A NanoCT scanner (Bioscan, USA; Mediso, Hungary) was used for this purpose, as previously reported^15,43^. The equipment was set up to maintain the samples at temperatures below −140 °C during vitrified tissue analysis. Once the temperature was reached, the samples were transferred from liquid nitrogen to the insulating container and CT image acquisition was performed. Specimens were then subjected to the warming protocol and NanoCT images were acquired at RT. This investigation was conducted to evaluate the washes for DMSO removal by searching for any remaining CPA.

Acquisition parameters included a 106 mA current for a voltage of 65 kV, exposure time per projection of 500 ms, and 360 projections per rotation. Each image required 3 minutes for total acquisition and was reconstructed to a spatial resolution of 0.2 mm. For imaging, three different software programs were used, namely Nucline software for acquisition (Mediso, Hungary), IVS image processing software for reconstruction (Invicro, USA), and PMOD 3.7 for analysis (PMOD Technologies LLC, Switzerland). For image visualization, a PMOD cold scale was applied, where the lowest attenuation is represented by a dark blue color and the highest attenuation by intense red, passing through green, yellow and orange for intermediate attenuation values.

### Freeze-substitution

The freeze-substitution technique enables visualization of possible ice formation in frozen or vitrified samples. Its main advantage is allowing preservation of tissue morphology as it is prior to warming, by fixing and processing fragments at low temperatures^44^. To this end, our samples were removed from the liquid nitrogen and transferred to a temperature of −80 °C. The medium was replaced with a solution containing 1% osmium tetroxide in methanol, which replaces water in tissues when changed over a series of 5 days. The samples were kept at −20 °C for 24 hours, before being warmed to 4 °C and left for a further 2 hours. The ovarian fragments were then dehydrated in a propylene oxide solution and infiltrated with epoxy resin. Sections measuring 2.5 µm in thickness were obtained and stained with toluidine blue, before analysis by light microscopy.

### Transmission electron microscopy

After fixation in Karnovsky’s solution, the samples were processed as previously described^45^. Ultrastructural organization was evaluated only in fresh controls and tissue obtained from 3 patients soon after warming, which showed higher follicle density. Post-fixation was achieved with 1% osmium tetroxide and contrast was performed with 0.5% uranyl acetate. A solution of 0.1 M cacodylate buffer was used to wash the tissue between steps. Increasing concentrations of ethanol were utilized to dehydrate the tissue before its inclusion in epoxy resin (Agar Scientific, Essex, UK). The blocks were subjected to semi-thin sectioning (3 µm), stained with toluidine blue, and analyzed by light microscopy to localize follicles. Thereafter ultrathin sections (70–90 nm) were obtained and analyzed by TEM (Zeiss, Germany). For TEM evaluation, the morphological characteristics of the stroma, granulosa cells and oocytes, as well as their organelles, basal and plasmatic membranes and nuclear envelope, were taken into account. Shape, morphology, distribution and the electron density of chromatin were also investigated.

## Data Availability

All data generated or analyzed during this study are included in this published article.
